# Automatic group-wise whole-brain short association fiber bundle labeling based on clustering and cortical surface information

**DOI:** 10.1186/s12938-020-00786-z

**Published:** 2020-06-03

**Authors:** Andrea Vázquez, Narciso López-López, Josselin Houenou, Cyril Poupon, Jean-François Mangin, Susana Ladra, Pamela Guevara

**Affiliations:** 1 Faculty of Engineering, Universidad de Concepción, Concepción, Chile; 2 Centro de investigación CITIC, Universidade da Coruña, A Coruña, Spain; 3 NeuroSpin, CEA, Paris-Saclay University, Gif-sur-Yvette, France; 4 INSERM U955 Unit, Mondor Institute for Biomedical Research, Team 15 “Translational Psychiatry”, Créteil, France; 5 Fondation Fondamental, Créteil, France; 6 AP-HP, Department of Psychiatry and Addictology, Mondor University Hospitals, School of Medicine, DHU PePsy, Créteil, France

**Keywords:** Fiber labeling, Clustering, Fiber bundle, Tractography, Superficial white matter

## Abstract

**Background:**

Diffusion MRI is the preferred non-invasive in vivo modality for the study of brain white matter connections. Tractography datasets contain 3D streamlines that can be analyzed to study the main brain white matter tracts. Fiber clustering methods have been used to automatically group similar fibers into clusters. However, due to inter-subject variability and artifacts, the resulting clusters are difficult to process for finding common connections across subjects, specially for superficial white matter.

**Methods:**

We present an automatic method for labeling of short association bundles on a group of subjects. The method is based on an intra-subject fiber clustering that generates compact fiber clusters. Posteriorly, the clusters are labeled based on the cortical connectivity of the fibers, taking as reference the Desikan–Killiany atlas, and named according to their relative position along one axis. Finally, two different strategies were applied and compared for the labeling of inter-subject bundles: a matching with the Hungarian algorithm, and a well-known fiber clustering algorithm, called QuickBundles.

**Results:**

Individual labeling was executed over four subjects, with an execution time of 3.6 min. An inspection of individual labeling based on a distance measure showed good correspondence among the four tested subjects. Two inter-subject labeling were successfully implemented and applied to 20 subjects and compared using a set of distance thresholds, ranging from a conservative value of 10 mm to a moderate value of 21 mm. Hungarian algorithm led to a high correspondence, but low reproducibility for all the thresholds, with 96 s of execution time. QuickBundles led to better correspondence, reproducibility and short execution time of 9 s. Hence, the whole processing for the inter-subject labeling over 20 subjects takes 1.17 h.

**Conclusion:**

We implemented a method for the automatic labeling of short bundles in individuals, based on an intra-subject clustering and the connectivity of the clusters with the cortex. The labels provide useful information for the visualization and analysis of individual connections, which is very difficult without any additional information. Furthermore, we provide two fast inter-subject bundle labeling methods. The obtained clusters could be used for performing manual or automatic connectivity analysis in individuals or across subjects.

## Background

The preferred technique to non-invasively study structural brain connections is diffusion-weighted magnetic resonance imaging (dMRI), based on the measurement of water molecules movement [1, 2]. Diffusion tractography estimates the main white matter (WM) tracts, obtaining a set of 3D paths, called streamlines or fibers [3]. Tractography datasets contain a large number of streamlines, some of which represent the trajectory of known WM bundles, with anatomical meaning. Such bundles have been described in the literature by neuroanatomists [4], and have been validated with other techniques like post-mortem dissections [5]. However, these datasets also include artifacts or false positives, some of which can occur systematically across subjects [6]. Hence, tractography datasets can be analyzed to extract or segment known WM bundles, which requires the inclusion of anatomical information in the processing. One strategy can be the manual delineation of regions of interest (ROIs) in the cortex, and the extraction of fibers connecting a pair of cortical regions for a specific bundle. This analysis has been recently used to study short association bundles [7]. The bundle segmentation can be performed automatically by applying an atlas of gray matter and WM ROIs, and then using anatomical descriptions of the bundles to segment fibers connecting or passing through specific ROIs [8].

Automatic methods based on ROIs allow an easy modification or addition of bundle extraction rules, but do not include an analysis based on the trajectories of the fibers as a whole. Another strategy is based on clustering to group fibers with similar shape and position, commonly based on a fiber pairwise distance measure that considers the Euclidean distances between the corresponding points (or closest points) of the two fibers. To extract anatomical bundles, some methods use a clustering algorithm and an atlas embedding anatomical bundle information [9–11]. Also, other simpler algorithms have been implemented to extract bundles based on a multi-subject bundle atlas [12–14]. Several atlases have been created to represent main deep white matter (DWM) bundles, which have been well described by anatomists and are very stable across subjects [9, 12, 15], i.e., present high similarity and can be found in all the subjects on medium- to high-quality databases. However, there exist several WM fiber bundles still unknown or not sufficiently described, because of their higher inter-subject variability and fewer reproducibility [16]. This is the case of short association bundles, where only a few works have been focused on their description for the whole-brain [17, 18]. Short association fibers are placed immediately underneath the gray matter of the cortex and connect adjacent or close gyri. They can present different sizes, where the shortest ones are the nearest to the cortex and present the typical U-shape, due to their closeness to the walls of the convolution depression [19]. Their description is still incomplete [16], however, post-mortem dissections have been used to validate the largest and reproducible bundles [7, 20]. Superficial white matter (SWM) fibers can be studied using exploratory fiber clustering methods that aim to detect fiber tracts without having any reference to the start or end of WM fibers [21]. This type of algorithm, applied to a whole-brain tractography dataset, generates a set of fiber clusters representing the main WM connections in the analyzed brain. In the case of the works in [17, 18], SWM bundle atlases were obtained using different methods based on fiber clustering and the addition of anatomical information. Also, a recent work found a great amount of SWM bundles [22], but those were not labeled, requiring a posterior analysis for their study. Hence, existing methods have been focused on finding reproducible bundles across subjects, but not on the development of an automatic labeling of individual or inter-subject SWM clusters. Whole-brain fiber clustering methods, applied to individuals or to a population of subjects, do not return directly the identification of the obtained clusters, e.g., information about the anatomical areas connected by the fibers and their relative position in the cortex. Such identification or labeling could be very useful for the study of the human brain connectome in individuals and different populations. The labeled clusters could then be used to perform detailed analyses of known bundles, i.e., subdivisions of the main bundles, and also of unknown fascicles, such as short association bundles. Furthermore, multi-subject analyses could be applied to create new bundle atlases.

Superficial white matter bundles are more variable across subjects and more susceptible to noise than deep white matter bundles, due to their smaller size and location in the brain, which presents partial volume effect. Hence special attention must be given to the diffusion local model and tractography methods. Due to the improvement of imaging quality, i.e., more signal-to-noise ratio, higher resolution, better distortion correction methods, between others, have allowed a better reconstruction of short association bundles. The most stable bundles can be reconstructed using deterministic tractography, with adapted parameters, in particular, a larger number of streamlines and a low FA threshold or an adapted propagation mask, to prevent the removal of voxels in the superficial white matter [16].

We propose a method that automatically labels the clusters of a subject obtained from an intra-subject clustering, based on the regions connected by the clusters. This information is based on a cortical surface mesh, labeled with the Desikan–Killiany atlas (35 gyri per hemisphere). Direct correspondence between subjects is obtained for the connected anatomical regions. Furthermore, within each region, the clusters on individuals are labeled following and ordering criterion. Moreover, we apply two strategies for inter-subject cluster labeling. First, a matching method is implemented based on the Hungarian algorithm to find correspondence between bundles across subjects and subsequently apply a labeling that gives the same names to bundles identified in several subjects. Also, a clustering algorithm is applied to group similar bundles on a set of subjects and perform the labeling across them. While the matching algorithm finds the best matching for single bundles, the clustering may group similar bundles on some subjects and identify similar bundles (or groups of bundles) across subjects. Both inter-subject implementations are fast, taking, respectively, about 96 and 9 s, respectively, over 20 subjects. The performance of both implementations was compared in terms of reproducibility and inter-subject bundle distance. The methods are publicly available from [23].

## Results

The experiments were executed on a computer with 4-core Intel Core i5-8250U CPU running at 1.60 GHz, 6MB of cache and 8GB of RAM, using Ubuntu 18.04.2 LTS with kernel 4.15.0-64 (64 bits). The programming language used to develop almost all stages is Python 3.6. For the analysis, the tractographies, cortical meshes, and labels according to Desikan–Killiany atlas of 20 subjects were used. The intra-subject labeling was applied to all the subjects. First, the results for intra-subject labeling performed on four subjects are shown and analyzed. Next, the two inter-subject labeling methods, matching and clustering, were applied to the 20 subjects, using a set of distance thresholds, ranging from a conservative value of 10 mm to a moderate value of 21 mm. We use the minimum average direct-flip (MDF) distance (Eq. 2). It is defined as the mean Euclidean distance between corresponding points of two centroids. We use centroids resampled with 21 equidistant points. Since centroids (or fibers) can be ordered in memory in opposite directions, the distance must be calculated with centroids in both directions (direct and flipped order), and then the minimum value must be selected, what will correspond to the correct order. The distance threshold defines the degree of similarity considered between bundle centroids. The smaller, the more restrictive the analysis will be.

The reproducibility of the bundles was evaluated by counting the number of subjects that had each inter-subject bundle. The quality of the labeling was evaluated using a distance measure between bundles across subjects (MDF). Also, heatmaps are shown to have an insight on the reproducibility and variability in terms of the number of fibers of the most reproducible bundles, for a restrictive distance threshold of 12 mm. Finally, some examples of bundles are displayed for a visual inspection of the results. Hungarian algorithm led to a high correspondence, but low reproducibility for all the thresholds, with 96 s of execution time. QuickBundles led to better correspondence and reproducibility with short execution time, of only 9 s. Hence, the whole processing for the inter-subject labeling over 20 subjects takes on average 1.17 h. In the following, we detail the results.

### Intra-subject labeling

The intra-subject labeling was applied to the 20 subjects. The input data are the tractography datasets of each subject and a cortical mesh where each vertex is labeled with the corresponding gyri (according to Desikan–Killiany atlas). The method consists of four stages (see Fig. 13). First, a fiber clustering *(stage 1)*, is applied to the whole-brain tractography dataset, which returns a set of clusters of similar fibers. Secondly, a cluster filtering *(stage 2)* is performed, where small and long fiber clusters are discarded, keeping only fibers on a reasonable range for short association fibers. Next, a fiber intersection *(stage 3)* is executed, to determine the intersection of the fibers with the cortical mesh. Finally, a cluster labeling *(stage 4)* is applied, that labels each cluster according to the two most connected cortical regions, and an index indicating its relative order in the region.

The fiber clustering *(stage 1)* led to about 43,000 clusters per subject. For fiber filtering *(stage 2)*, a filter with a minimum cluster size of $min_{nf}=10$ fibers is used to discard small clusters. Also, a minimum cluster length of $min_{len}= 30\,\hbox {mm}$ and a maximum cluster length of $max_{len}= 80\,\hbox {mm}$ were employed to discard clusters that are too short or too long, leading to an average of 1100 clusters per subject. The filtering values are similar to those previously used [17, 18]. After applying fiber intersection and cluster labeling stages *(stage 3 and 4)*, the clusters of each subject were labeled according to the pair of anatomical regions connected by each bundle, and the position based on ascending order of *y*-axis on MNI space (default configuration), i.e., from the bottom up.

**Fig. 1:**
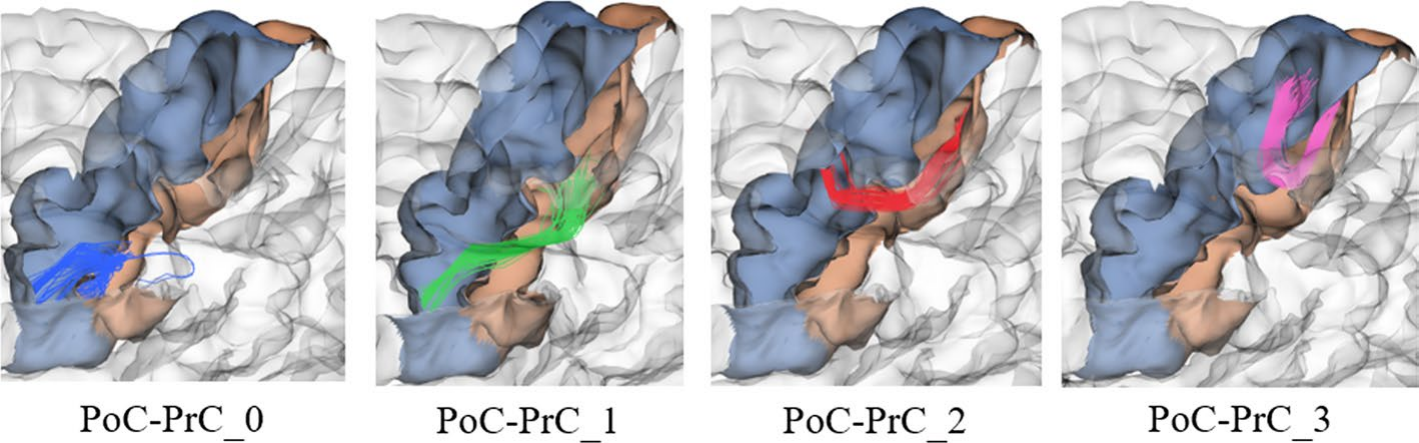
Bundles connecting right PoC and PrC regions. Example of bundle labeling according to the relative position of the bundles connecting *PoC* and *PrC* regions for Subject001

An example of the relative ordering for intra-subject bundle labeling is presented in Fig. 1. We can appreciate that bundles connecting *postcentral (PoC)* and *precentral (PrC)* regions are ordered according to *y*-axis in ascending order (from the bottom up). Bundles are ordered according to the *PoC* parcel since it is indexed before in the Desikan–Killiany atlas.

**Fig. 2:**
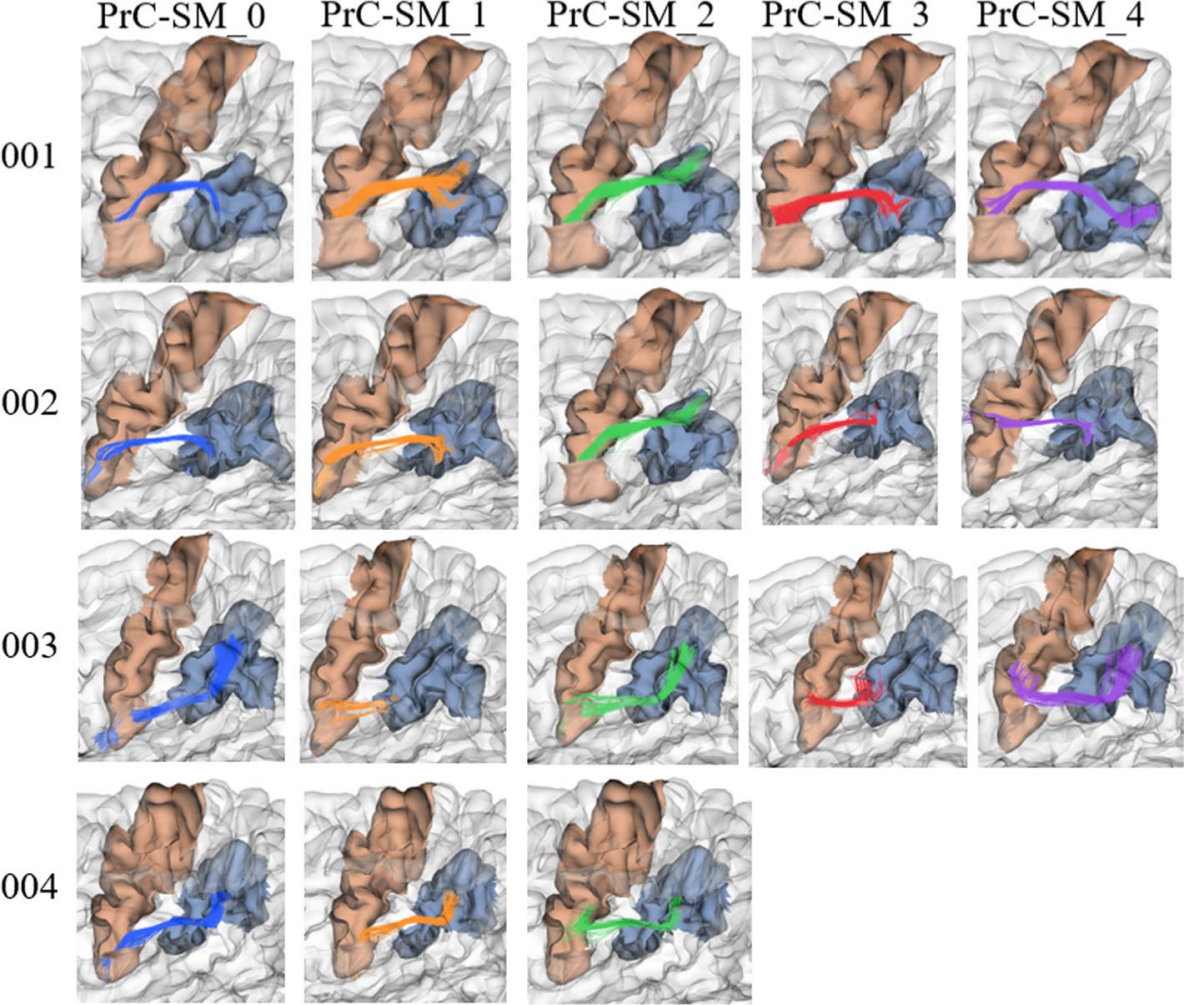
Correspondence of intra-subject bundle labels across subjects. Comparison of the first five bundles from four subjects (001–004), connecting *PrC* and *SM* gyri

Even though the method performs only an intra-subject analysis, a degree of correspondence between the four first subjects can be found, according to their relative position index. Because of inter-subject variability, the correspondence is not perfect, nor do they all have the same number of bundles. Figure 2 displays the first five bundles of the four subjects, which connect the left *PrC* gyri with the *supra-marginal (SM)* gyri.

**Fig. 3:**
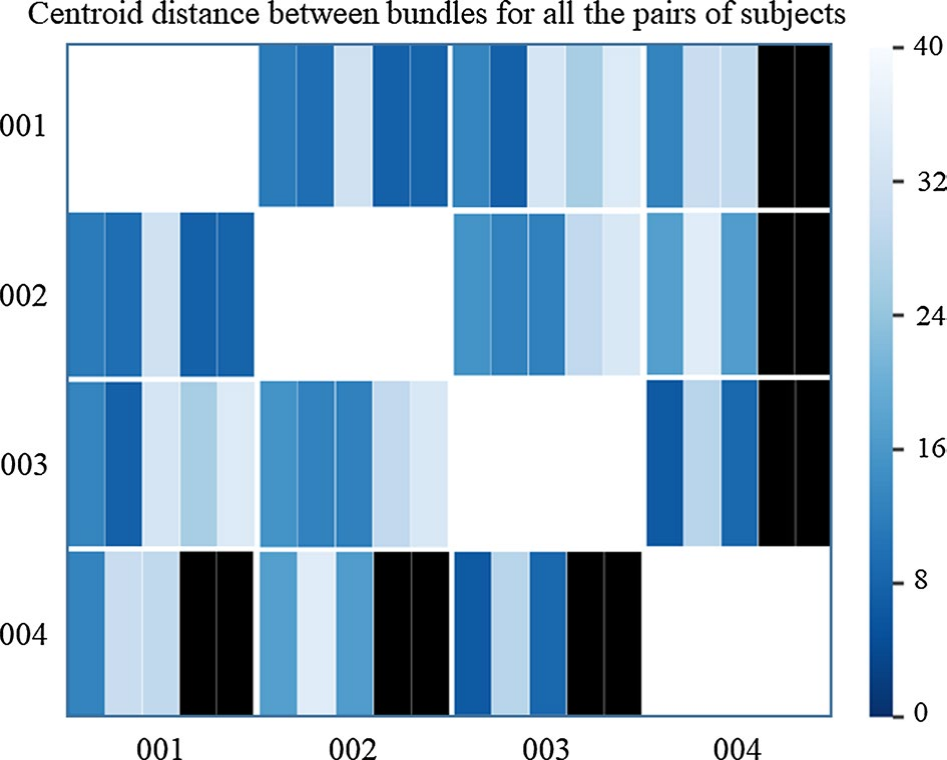
Bundle centroid distances between pairs of subjects for intra-subject labeling of four subjects. Each cell in the matrix contains five divisions. Each division represents a bundle connecting *PrC* and *SM* gyri (*PrC_SM_0* to *PrC_SM_4*). The color scale represents the distances (in mm) between bundle centroids for all the pairs of subjects. The black divisions represent the absence of bundles connecting the gyri

A quantitative evaluation of the bundle correspondence among subjects is displayed in Fig. 3, where the distance (MDF) between the bundle centroids of each pair of subjects for the five bundles connecting *PrC* and *SM* gyri (*PrC_SM_0* to *PrC_SM_4*) is represented with a color scale in mm. Bundles show a relatively good correspondence among them, with distances between centroids ranging from 7 to 36 mm, with an average of about 20 mm. Note that distances of 20–30 mm have been previously used for inter-subject analyses of superficial white matter [17, 18].

Finally, the average execution times for each stage of the intra-subject labeling are: 192 s for *stage 1*, 10.23 s for *stage 2*, 11 s for *stage 3* and 2.49 s for *stage 4*, taking on average a total time of 3.6 min.

### Inter-subject labeling

The inter-subject labeling was applied to 20 subjects from the ARCHI database [24]. A comparison has been made between the two implemented methods, matching with the *Hungarian* algorithm [25] and clustering with *QuickBundles (QB)* algorithm [26]. Both algorithms work with an input parameter, a distance threshold, using the minimum average direct-flip (MDF) distance [26] from one centroid to another. This distance threshold defines the minimum degree of similarity between bundles. In addition, tests have been carried out with four different distance thresholds. The first threshold of 10 mm is very conservative, being the default threshold of *QB* for intra-subject clustering. We also used a 12 mm threshold, which is still conservative and aims to find similar bundles across subjects. Two other moderate thresholds were used: 18 mm and 21 mm, which are adequate considering that distances of 20–30 mm have been previously used for SWM inter-subject analyses [17, 18].

**Fig. 4:**
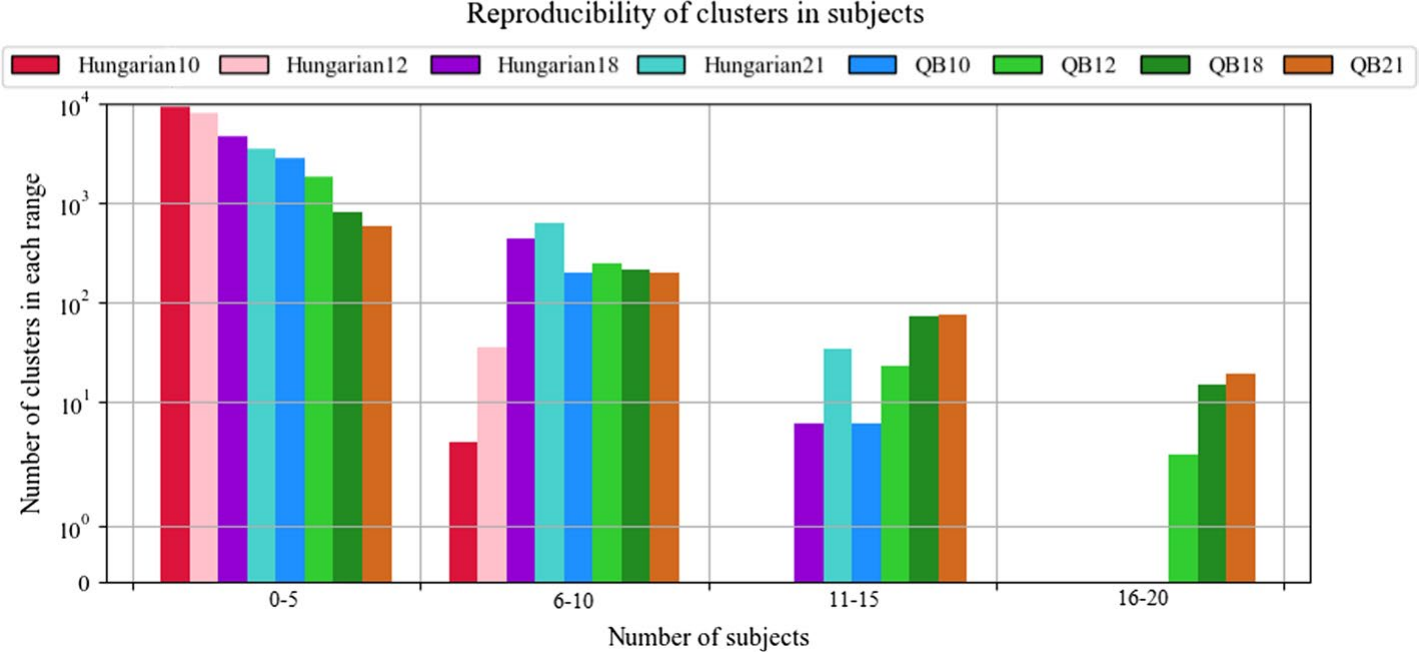
Reproducibility of bundles with inter-subject labeling for the two methods. The number of subjects is shown on the *x*-axis while the *y*-axis shows the number of clusters in each range

The reproducibility of the methods was evaluated by counting the number of subjects in which each bundle was found. Figure 4 shows the reproducibility of the bundles for both methods and the four thresholds. Table 1 lists three reproducibility indices for the two inter-subject labeling methods, separated by hemisphere: the maximum number of subjects for the bundles within the 20 most reproducible bundles, and the number of bundles with reproducibility greater than or equal to 50% and 75%. As expected, for both algorithms, the higher the distance threshold, the greater the reproducibility. As can be seen, the method that shows the highest reproducibility is *QB*, presenting 94 bundles with more than 50% of reproducibility for a distance threshold of 21 mm, which is a good number, based on previous studies [17, 18]. On the other hand, the *Hungarian* algorithm only found 34 bundles with more than 50% of reproducibility for the same threshold. Furthermore, the *Hungarian* algorithm found no bundles present in all subjects, while *QB* found 19 for 21 mm threshold. As the *Hungarian* algorithm tries to match 1–1 the bundles, leads to less reproducibility than *QB*.

**Table 1:** Reproducibility values between for the two inter-subject labeling methods

Method	Max	# Bundles $\ge 50\%$	# Bundles $\ge 75\%$
Hungarian12_left	7	0	0
Hungarian12_right	6	0	0
QB12_left	19	12	3
QB12_right	14	14	0
Hungarian18_left	13	3	0
Hungarian18_right	11	3	0
QB18_left	20	41	9
QB18_right	19	42	6
Hungarian21_left	13	22	0
Hungarian21_right	13	12	0
QB21_left	20	49	10
QB21_right	20	45	9

The left column identifies the method (Hungarian or QB), hemisphere (left or right), and the thresholds (12 mm, 18 mm or 21 mm). The second column lists the maximum number of subjects for the bundles within the 20 most reproducible bundles. Columns three and four show the number of bundles with reproducibility greater than or equal to 50% and 75%, respectively

**Fig. 5:**
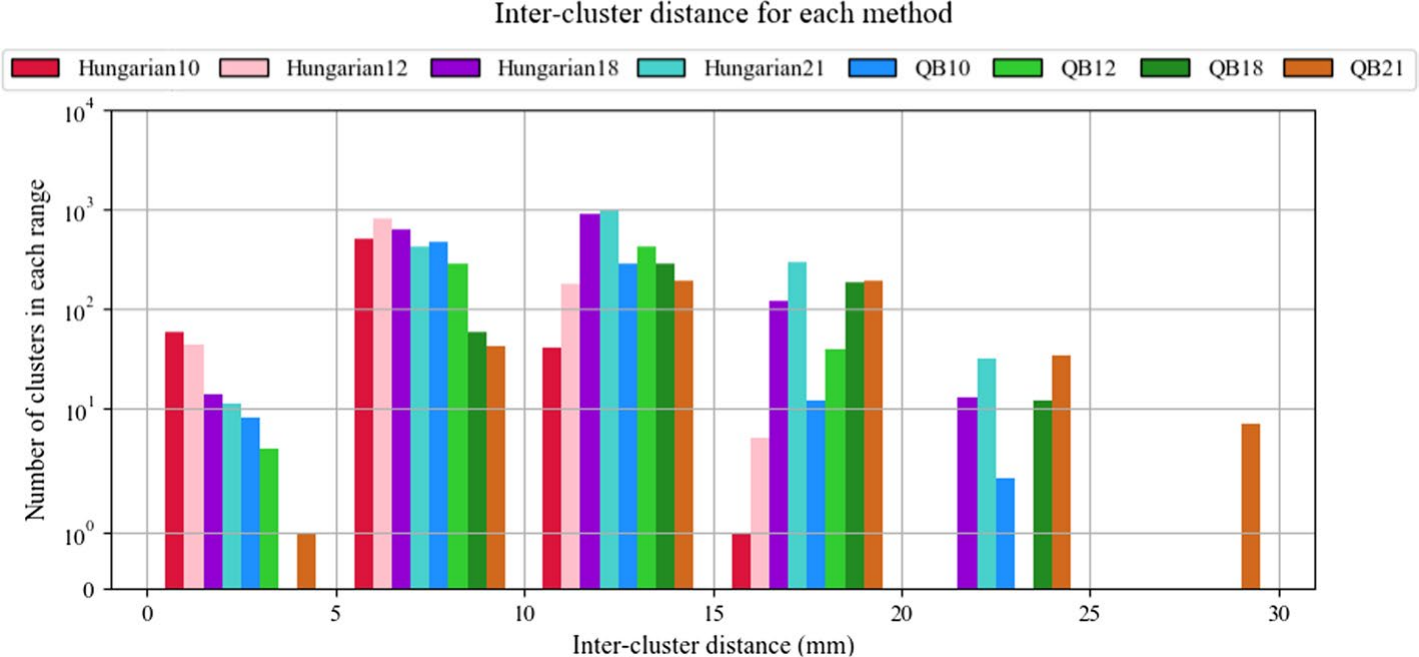
Inter-cluster bundle distance for both inter-subject labeling method. *X*-axis represents the inter-cluster distance measured in mm. *Y*-axis shows the number of clusters in each range

Figure 5 shows inter-subject labeling quality for both methods with the four tested distance thresholds. The quality is evaluated using the inter-cluster distance (MDF), calculated per each bundle as the average distance between all the pairs of bundle centroids from the subjects where the bundle was labeled. Thus, the clusters classified with the same label are measured together, the closer the clusters are in all the subjects, the better the quality of the method. As expected, for both algorithms, the lower the distance threshold, the higher the quality. It can be seen that the most accurate algorithm is the *Hungarian* with a 10-mm threshold, at expenses of a low reproducibility, as shown above. The *QB* algorithm has a lower quality than *Hungarian* because it groups clusters of the same subject and merges them, thus increasing the inter-cluster distance. However, the merging of close clusters leads to a final better reproducibility, while keeping a moderate intra-cluster distance across subjects, with values inferior to 30 mm, and an average of about 15 mm.

**Fig. 6:**
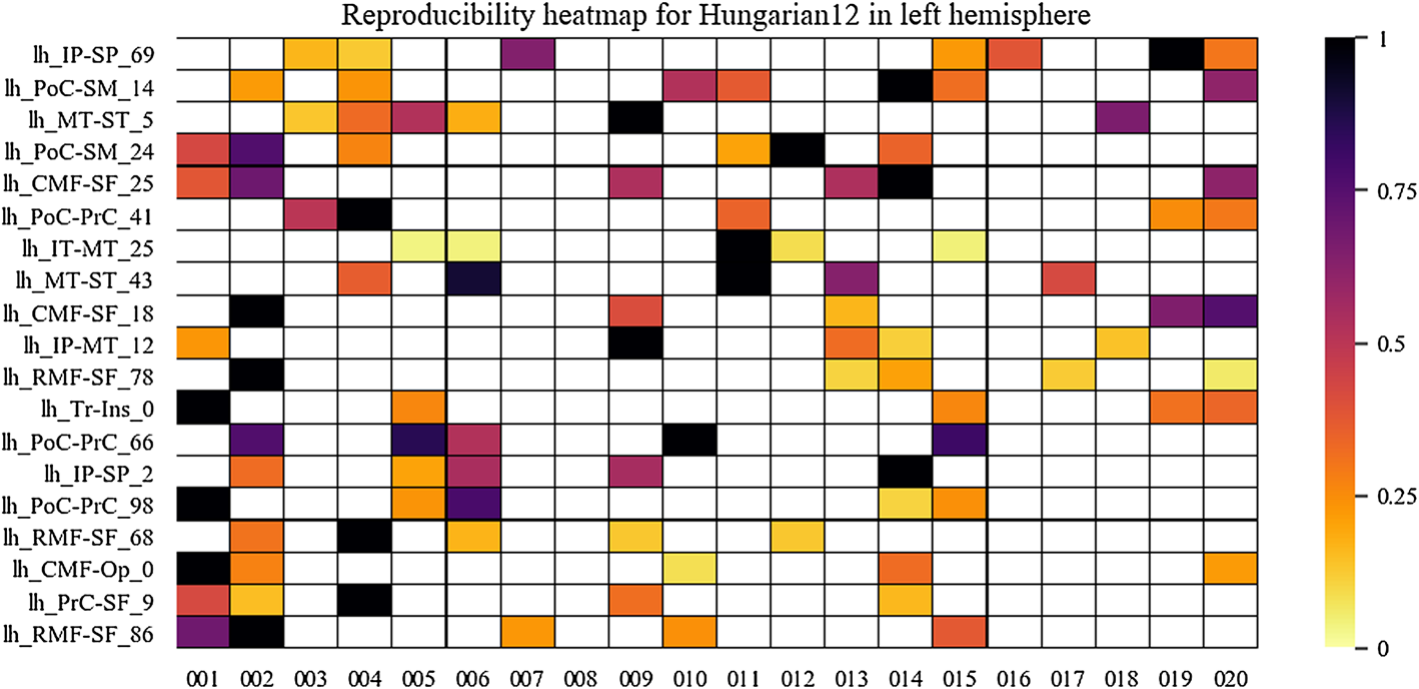
Reproducibility heatmap for Hungarian algorithm with threshold 12 mm, for the left hemisphere. On the *x*-axis are the subjects, on the *y*-axis are the 20 most reproducible bundles. The greater the number of fibers, the darker the color of the box on the heatmap that is normalized between 0 and 1

**Fig. 7:**
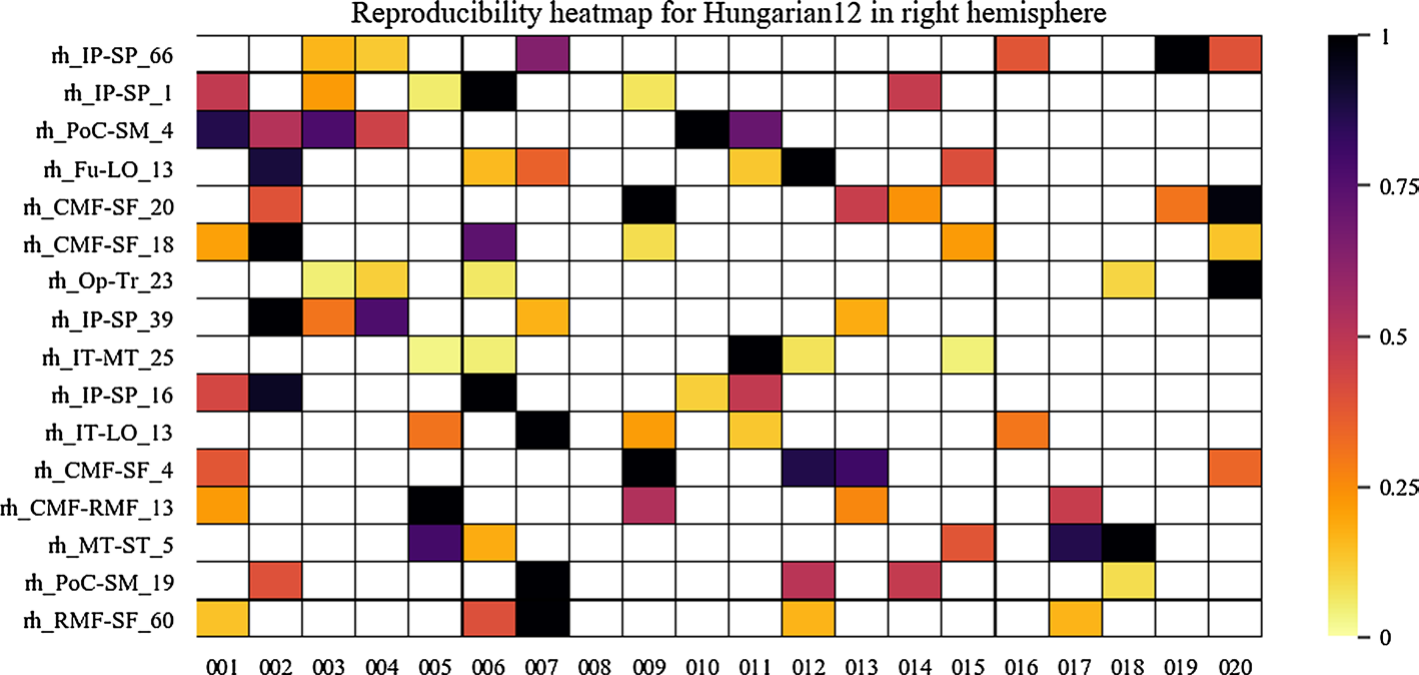
Reproducibility heatmap for Hungarian algorithm with threshold 12 mm, for the right hemisphere. *X*-axis displays the subjects used, the 20 most reproducible bundles are shown on the *y*-axis. The darker boxes indicate a higher concentration of fibers in the bundle. These values are normalized between 0 and 1

**Fig. 8:**
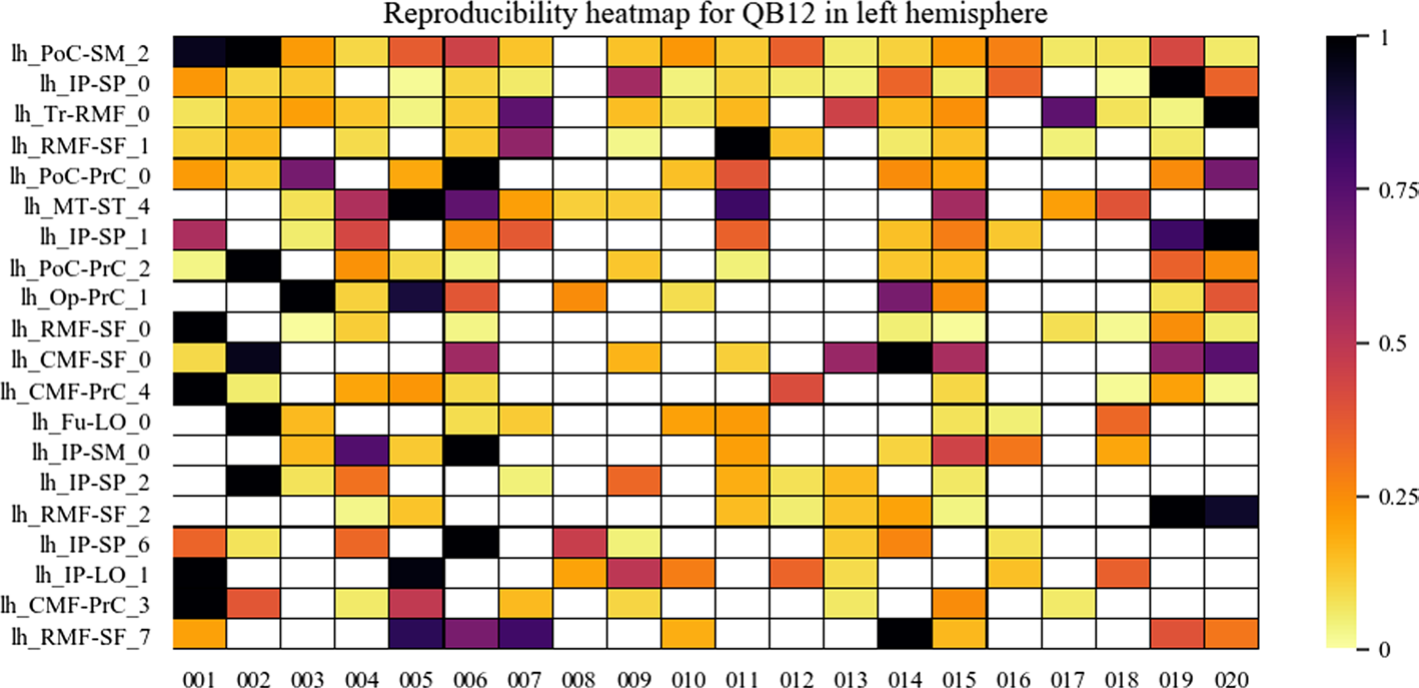
Reproducibility heatmap for QB with threshold 12 mm, for the left hemisphere. *X*-axis shows the subjects, while the *y*-axis shows the 20 most reproducible bundles among subjects in the left hemisphere. Darker boxes show bundles with more fibers in them. White boxes show the absence of the bundle in the determined subject. The heat bar shows the values of normalized fibers between 0 and 1

**Fig. 9:**
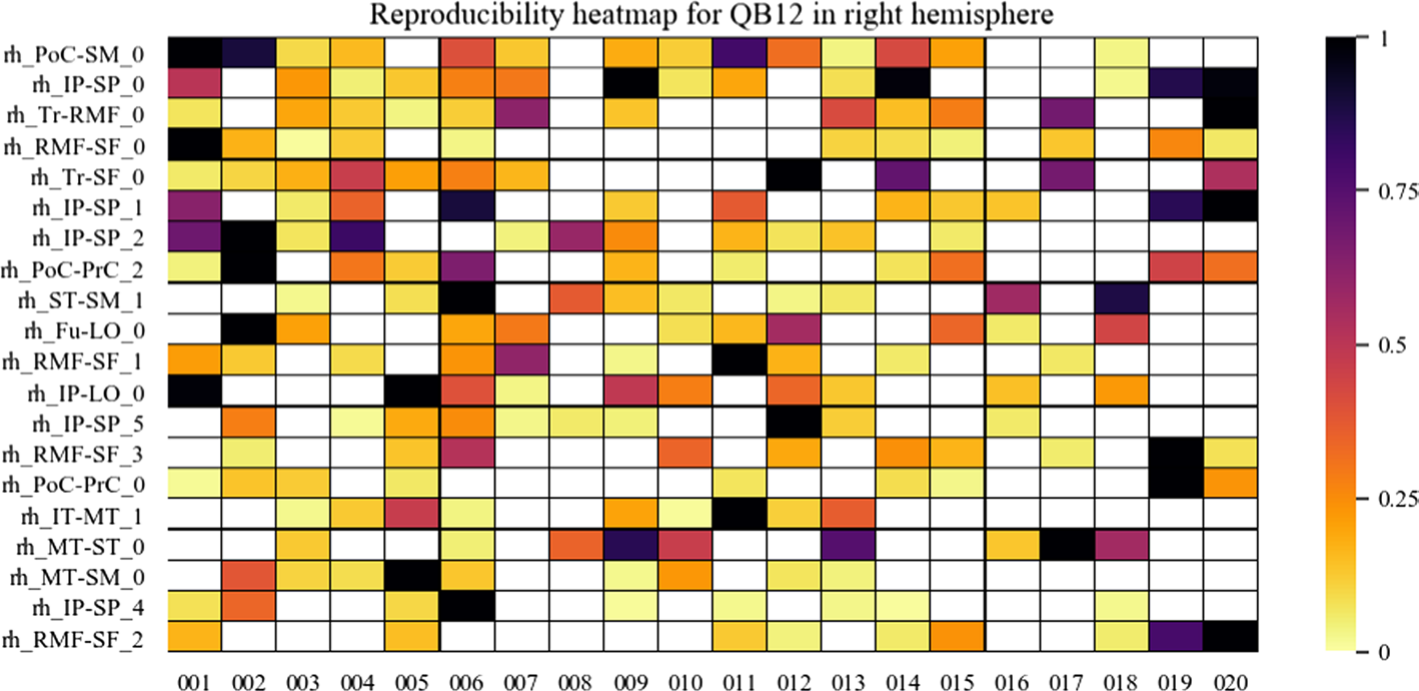
Reproducibility heatmap for QB with threshold 12 mm, for the right hemisphere. *X*-axis displays the subjects, and on the *y*-axis appears the 20 most reproducible bundles. The lighter the color of the box, the fewer fibers it contains. If the box is white, it indicates the absence of the bundle in the subject. The fiber values appear normalized between 0 and 1 in the heat bar

To have an insight of the reproducibility and variability of the most reproducible bundles in all subjects for the two labeling methods, we created heatmaps (Figs. 6, 7, 8 and 9). The heatmaps were created separately for the 20 bundles of the left and right hemispheres with the highest reproducibility in the 20 subjects, for a 12 mm threshold. Figures 6 and 7 display the heatmaps for the *Hungarian* algorithm, for left and right hemispheres, respectively, while Figs. 8 and 9 show the heatmaps for the *QB* clustering algorithm. The bundles appear in descending order along the *y*-axis, according to the reproducibility between subjects, which appear along the *x*-axis. Empty (white) boxes indicate that a bundle does not exist in a certain subject. The colors represent the normalized number of fibers of each bundle (0–1), the darker, more fibers.

It can be seen that, as in Fig. 5, the method with the highest reproducibility is *QB*. The number of fibers seems to be more homogeneous for *QB*, with a tendency of a low normalized number of fibers. This does not mean that the bundles have few fibers, but that their number is of a given value for most of the subjects, with very high values for a few subjects. The bundle with the highest reproducibility is *lh_PoC-SM_2* which was found in 19 subjects, followed by *lh_IP-SP_0* and *lh_Tr-RMF_0*, both found in 18 subjects. Reproducibility using matching is poorer, whose most reproducible bundle, *lh_IP-SP_69*, appears in only seven subjects.

**Fig. 10:**
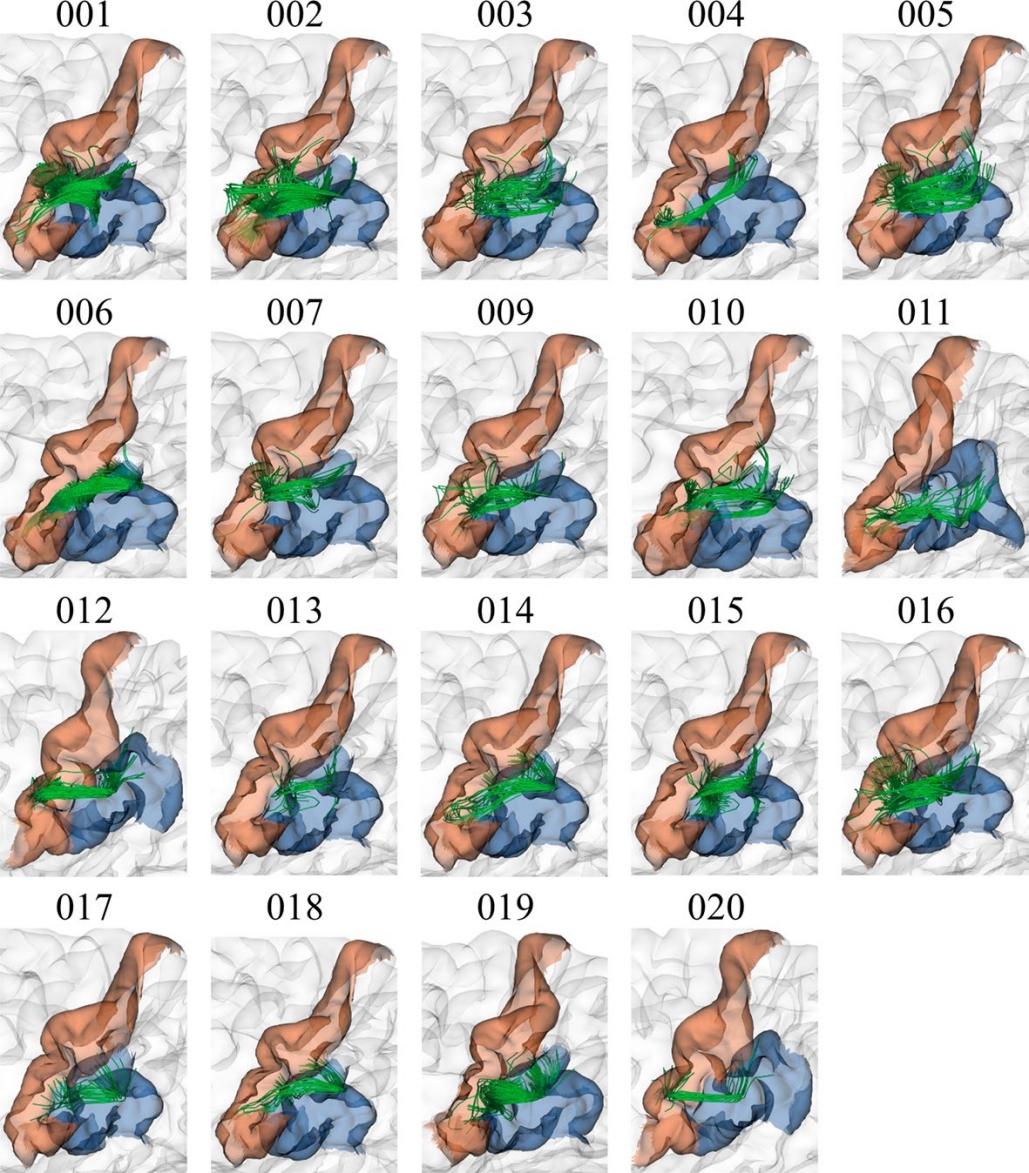
Bundle lh_PoC-SM_2, with the highest reproducibility in all subjects. The results show good reproducibility among subjects, appearing in 19 of the 20 subjects for the *QB* method with 12 mm threshold

Finally, some examples of bundles with high, medium and low reproducibility are displayed for a visual inspection of the results. Figure 10 shows the bundle *lh_PoC-SM_2*, belonging to the left hemisphere and classified by the *QB* clustering method with a 12 mm threshold. This is the bundle with the highest reproducibility with this restrictive threshold, being present in 19 out of the 20 subjects, with the exception of *Subject008*, achieving a 95% of reproducibility. It can be seen how bundles connect approximately the same cortical regions in different subjects and have a similar main shape. Also, it can be seen that the number of fibers is very variable among subjects, which is usual in SWM bundles.

**Fig. 11:**
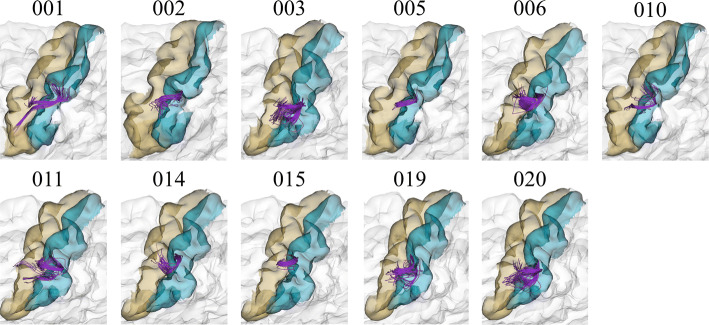
Bundle PoC-PrC_0, with medium reproducibility. The *PoC-PrC_0* bundle appears in 11 out of 20 subjects, achieving 55% of reproducibility, for *QB* algorithm with a 12 mm threshold

Figure 11 shows the bundle *lh_PoC-PrC_0*, of the left hemisphere and classified by the *QB* method with 12 mm of threshold. It appeared in 11 out of the 20 subjects, that is, a 55% of reproducibility. This is a small bundle connecting the *PoC* and *PrC* gyri.

**Fig. 12:**
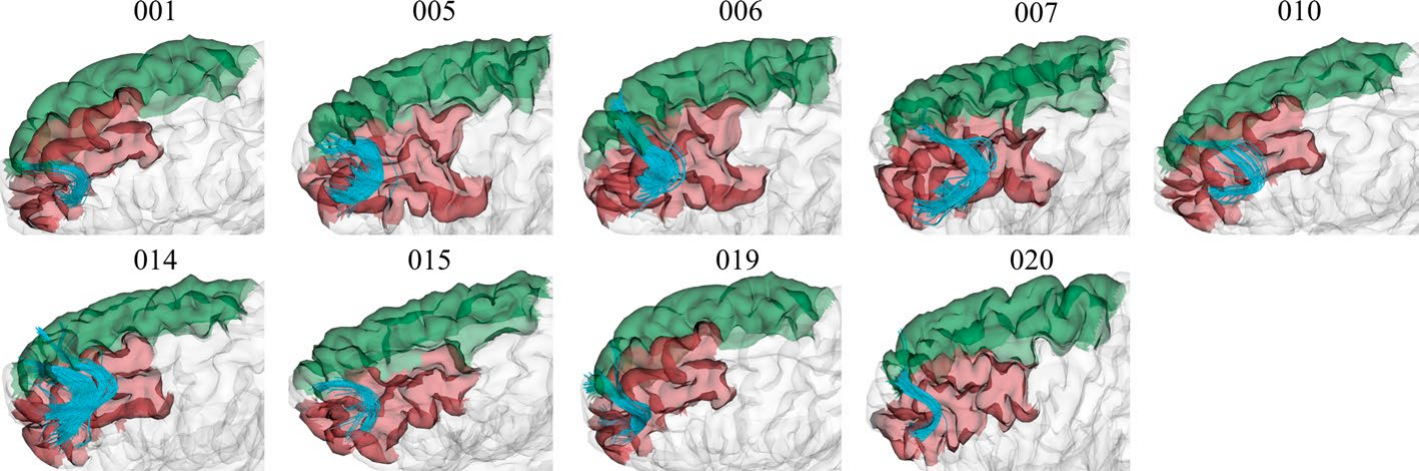
Bundle lh_RMF-SF_7, with low reproducibility. The bundle appears in nine out of 20 subjects using the *QB* clustering algorithm with a 12 mm threshold

Lastly, Fig. 12 shows for the left hemisphere the cluster *lh_RMF-SF_7*, classified by the *QB* method using a threshold of 12 mm. This is the least reproducible cluster of the heatmap of Fig. 8, appearing in 9 out of the 20 subjects, reaching only 45% of reproducibility. However, it can be seen that the bundles connect the same area in all subjects, slightly varying the position and the number of fibers.

## Discussion

In the last two decades, a great number of methods have been proposed for the analysis of tractography datasets. Most of the works have been focused on the study of deep white matter bundles, such as the arcuate fasciculus or the inferior fronto-occipital fasciculus. These bundles are in general larger and more stable across subjects, and have been described by neuroanatomists several decades ago. The methods have been focused on the study of these bundles, the creation of WM bundle atlases and the segmentation of WM bundles. Most of the studies have been developed with a combination of ROI-based and clustering-based methods, and the important guidance of neuroanatomy experts. In general, the applications analyze the segmented bundles across subjects and different populations of patients.

The methods have evolved with the increasing quality of the data. Tractography datasets have increased their size and complexity due to a higher resolution and better image quality, being able to provide a better representation of fiber crossing and small bundles. These advances are also associated with improved algorithms along all the processing pipeline, including artifacts and distortion corrections, diffusion local modeling and fiber tracking. Furthermore, the use of more accurate tractography propagation masks (e.g., based on T1 images [27]) has helped to achieve a better reconstruction of small and superficial white matter fibers.

Hence, in the last decade, due to the better quality of dMRI images and processing algorithms, it has been possible to start studying the short association WM bundles. A first whole-brain study used an atlas of gray and white matter to extract short fibers connecting adjacent gyri [28]. Other works combined a hierarchical fiber clustering and cortical parcellation information to extract reproducible short association bundles [17]. A recent study reported a great number of short association bundles, but without a labeling [22]. These works were mostly focused on the creation of SWM atlases. Hence, there is still a need for methods, open to the research community for the study of short association bundles in new databases.

The proposed methods provide an automatic labeling of SMW bundles. First, an efficient individual labeling was implemented. It generates compact clusters and labels them according to a cortical parcellation based on mesh information, for a high-quality ROI-based labeling. Furthermore, the bundles connecting each pair of anatomical regions (gyri) are ordered following an axis orientation. The resulting clusters could be used for fast and easy exploration of short association bundles in individual brains. Without a labeling, its exploration is very complex, since about one thousand clusters are produced for the whole brain.

Subsequently, an inter-subject method has been added, to obtain a correspondence between the clusters (or bundles) across subjects. We tested two methods, a matching, based on the *Hungarian* algorithm, and a clustering method, based on the *QB* algorithm. Even though we used available implementations of both methods, we have adapted them to the processing of labeled intra-subject clusters from different subjects to generate automatically labeled inter-subject clusters.

Since there is a high inter-subject variability in tractography datasets, especially for short association bundles, the applied processing extracts the main fiber connections by using first an intra-subject clustering. It has also the advantage to remove noisy fibers, that are not grouped into the main clusters. Furthermore, the inter-subject analysis, along with a common labeling for clusters which are similar for most of the subjects, performs an identification of non-reproducible fibers at the group level.

The results show a better reproducibility across subjects for the *QB* clustering method versus the *Hungarian* algorithm. *Hungarian* algorithm finds a good correspondence between subjects, with low inter-cluster distance, but at expenses of inferior reproducibility. Due to inter-subject variability, and the absence of bundles in some subjects, the one-to-one matching strategy seems not to be directly applicable to this kind of problem. On the other side, the clustering groups similar bundles on subjects and do not impose the existence of clusters in all the subjects. Indeed previous inter-subject analyses based on clustering have included a reproducibility constraint, e.g., a minimum number of subjects present in the clusters. Hence, an advantage of the proposed labeling is that this reproducibility information is easily extracted from the inter-subject labels, which is not the case for classic algorithms. Furthermore, even though the main goal of this work is not a study of the reproducibility of SWM bundles, the results of our inter-subject clustering strategy are competitive with state-of-the-art methods, with 94 reproducible bundles for a moderate MDF distance of 21 mm, compared to about 100 hundred bundles obtained for atlases proposed in [17, 18], created with a maximum Euclidean distance of 30 mm.

Note that several factors impact the results, including the quality of the tractography datasets, and the registration strategy. It has been shown that using non-linear registration increases the number of SWM identified [18]. In our experiments we used affine registration to Talairach space, however, other registration algorithms can be applied without problem. Furthermore, false positives are very likely to increase the variability across subjects and affect the results. Bundle variability may also be due to the inherent variability of the cortical folding patterns [29]. Since U-fibers are directly under the cortical sulci, present a smaller size, and connect small regions of the cortex, they are more sensitive to differences in the cortex anatomy than deep white matter bundles.

Finally, we highlight some advantages of the proposed methods. First, it is efficient, taking about 3.6 min for an intra-subject analysis and about 9 s to perform the inter-subject clustering. That is, for the whole inter-subject labeling processing, it takes about 1.17 h on average. This time is reasonable for an inter-subject analysis. Furthermore, the algorithms are scalable and can be applied to larger tractography datasets and databases.

Inter-subject labeling can be used to discover patterns of connections in different groups of healthy subjects and patients. The inter-subject clusters can be used to create WM bundle atlases, which require the inspection of experts in anatomy. Note that the clusters from tractography can contain artifacts and false positives [6]. In fact, false-positives can be highly reproducible, hence these will be also labeled by the proposed methods. The inter-subject labeling was not conceived to identify bundles with an anatomical meaning or to discard false-positives. The objective is to identify and label all the reproducible bundles. These bundles can be used as input to other analyses that include anatomical knowledge, in order to validate the bundles. The proposed algorithm can also contribute to the analysis of tractography datasets for the improvement of tractography methods, through the incorporation of anatomical information and filtering. Finally, other applications include the study of brain connectomes and methods for diffusion-based cortical parcellations.

## Conclusions

We implemented a fast method for the automatic labeling of white matter fiber bundles, specifically for the *SWM*, based on an intra-subject clustering and the connectivity of the clusters with the cortical mesh, based on an anatomical ROI atlas. The algorithm also adds a label associated with the relative position of the bundles. Results for intra-subject labeling show a degree of correspondence between subjects, which is further improved with inter-subject labeling. A complete intra-subject labeling is executed on an average time of 3 min and 35 s for a tractography dataset of about one million fibers. This enables a fast and easy exploration, visualization and analysis of labeled short association bundles in individuals, which is very difficult without any additional information.

Besides, we developed an inter-subject labeling by using two methods. One approach is matching, in particular, the *Hungarian* algorithm, and the other is clustering, employing *QuickBundles* algorithm. The results show a better reproducibility across subjects for the clustering method versus the matching algorithm while keeping a moderate inter-cluster distance, indicating a good quality of the clusters. Furthermore, the algorithm is scalable and the whole processing for the inter-subject labeling executes at a reasonable time, of about 1.17 h for 20 subjects. The obtained clusters could be used to perform group-wise connectivity studies, such as the creation of WM bundle atlases, and the development of new methods for the analysis of brain connectome.

Future work will be focused on the application of the method in high-quality databases, such as the *Human Connectome Project (HCP)* database, for the creation of a SWM atlas and diffusion-based cortical parcellations.

## Methods

### Database and tractography datasets

We used 20 subjects of the ARCHI database [24], which was acquired with a 3T MRI scanner (Siemens, Erlangen). The MRI protocol included the acquisition of a T1-weighted dataset using an MPRAGE sequence (160 slices; matrix = 256 $$\times$$ 240; voxel size = 1 $$\times$$ 1 $$\times$$ 1.1 mm) and a SS-EPI single-shell HARDI dataset along 60 optimized DW directions, $$\hbox {b}=1500$$$$\text {s/mm}^2$$ (70 slices, $$\hbox {TH}=1.7$$ mm, $$\hbox {TE}=93$$ ms, $$\hbox {TR}=14,000$$ ms, $$\hbox {FA}=90$$, matrix = 128 $$\times$$ 128, $$\hbox {RBW}=1502$$ Hz/pixel). BrainVISA/Connectomist 2.0 [30] was used for pre-processing the images. The HARDI dataset was corrected for artifacts, geometrical distortions induced by susceptibility effects, eddy currents, and motion. The diffusion-weighted (DW) processing pipeline includes the calculation of the analytic q-ball diffusion model [31]. Whole-brain streamline deterministic tractography [32] was calculated with one seed per voxel, from all the voxels of the T1-based propagation mask [27], in forward and backward directions, with a tracking step of 0.2 mm and a maximum curvature angle of $$30^{\circ }$$. Resulting tractography datasets contain about one million fibers per subject. These calculations were performed using Brainvisa/Connectomist software,^1^ however other software can be used, such as DSI Studio.^2^ Special attention must be given to the propagation mask, by the use of a low FA threshold and a visual inspection of the mask, to prevent the remotion of superficial white matter voxels. More details about tractography parameters for the reconstruction of superficial white matter bundles and their reproducibility can be found in [16].

Affine transformations between subjects’ T1 and DW images were also calculated, as well as affine transformations from T1 to Talairach space. The cortical meshes and an automatic labeling of the anatomical regions according to the Desikan–Killiany atlas were obtained using FreeSurfer [33].

### Automatic labeling of SWM bundles

**Fig. 13:**
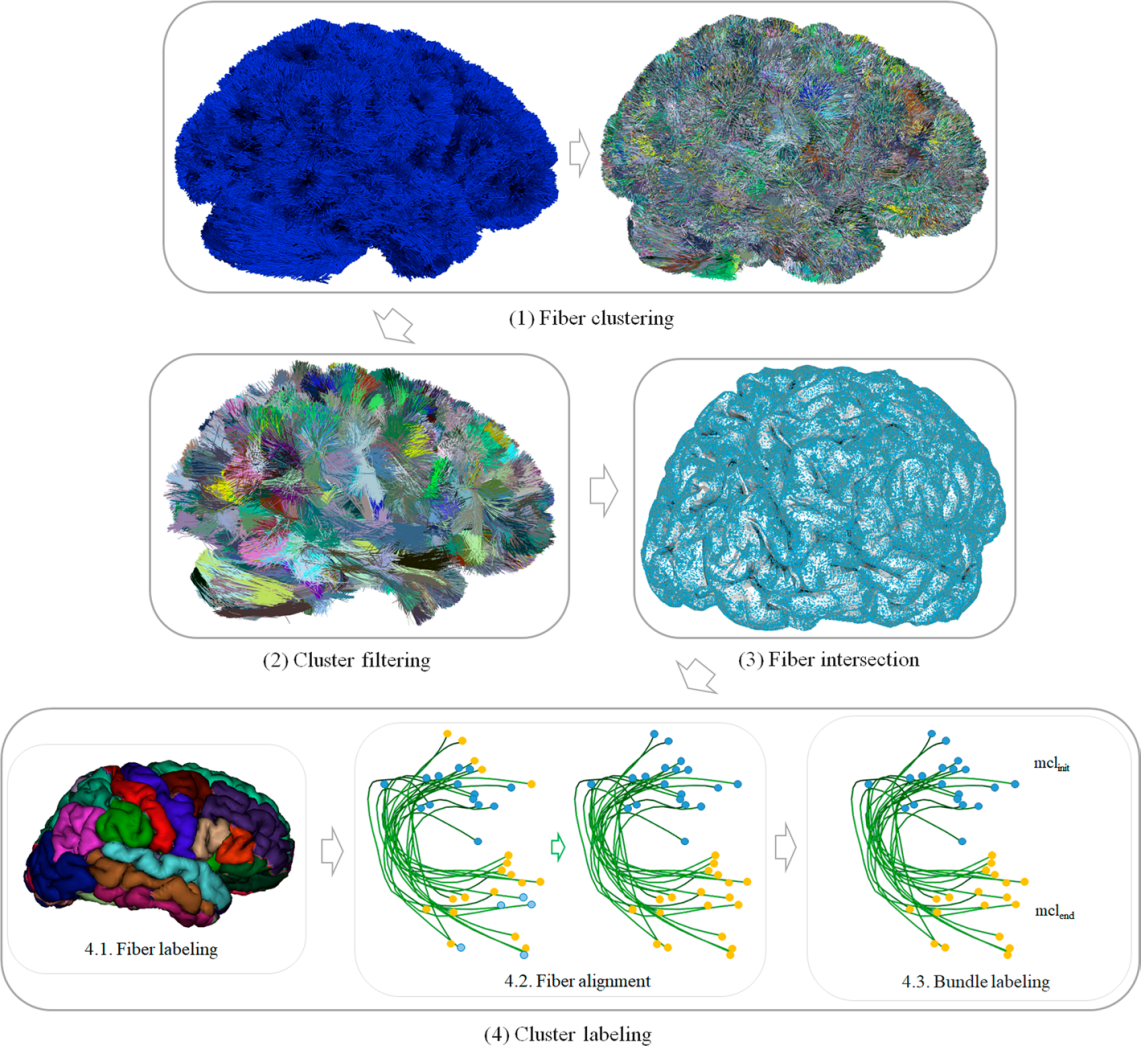
Schematics of the labeling method. Stage 1: fiber clustering. Performs the clustering of the entire tractography. Stage 2: cluster filtering. Filters out the small clusters and only keep the short bundles, obtained in the previous stage. Stage 3: fiber intersection. Calculates the fiber bundle intersection with the cortical mesh. Stage 4: cluster labeling. Renames the clusters based on the two connected regions of the cortex and their relative position

To perform the automatic labeling of bundles of superficial white matter, a method consisting of four stages (see Fig. 13) was developed, these are: *(1)* fiber clustering, *(2)* cluster filtering, *(3)* fiber intersection and *(4)* cluster labeling.

**Stage 1: fiber clustering**

This first stage performs an automatic clustering of a whole-brain tractography dataset, which returns a set of clusters of similar fibers (see Fig. 13-(1)). The clustering method [34] is an improved version of an algorithm proposed in [35]. To apply the clustering, fibers must be first resampled with 21 equidistant points, as in [12, 36]. The method consists of 4 steps: *(1) building clusters on a subset of fiber points*, where mini batch K-means is applied in parallel on a subset of fiber points, obtaining groups of points; *(2) generating preliminary clusters*, which groups fibers sharing the point cluster labels from the previous step; *(3) Defining candidate clusters by reassigning small preliminary clusters*: reassigns small clusters to larger clusters based on a maximum distance threshold between clusters; *(4) computing final clusters by merging close candidate clusters*: merges close clusters that share the central label obtained from step 1, according to a criterion of maximum Euclidean distance between clusters. Finally, a representative fiber of each cluster is selected, as its centroid, and resampled with 21 equidistant points.

**Stage 2: cluster filtering**

The second stage automatically filters out the small and long fiber clusters (see Fig. 13-(2)). Clusters are denoted as $C_i$, with $i = 1, ..., n$ the index of the cluster. The filter receives a minimum size of the cluster $min_{nf}(C_i)$ (number of fibers), to remove small fibers, and a minimum $min_{len}(C_i)$ and maximum cluster length $max_{len}(C_i)$ to only keep short fibers within a reasonable range. The length of each cluster is measured using the Euclidean distance between two adjacent points of the cluster’s centroid.

**Stage 3: fiber intersection**

This step automatically calculates the intersection of the fibers with the cortical mesh, based on the algorithm proposed in [37] (see Fig. 13-(3)). The method first performs a subdivision of 3*D* space into 1.5-mm-size cells, which speeds up searches in the mesh. Next, each fiber endpoint is projected one point backward and two points forward to extend the search along the fiber trajectory on ending points and avoid missing intersections. All cells that include these points and their neighboring cells are selected. Finally, the intersection point of each fiber extremity with the cortical mesh triangles is determined using Möller–Trumbore equation [38], based on the analysis of the triangles contained in the selected cells.

The intersection algorithm is given by Eq. 1:

1 $$\begin{aligned} O +tD = (1-u-v)V_0 + uV_1 + vV_2, \end{aligned}$$

where (*u*, *v*) are the exact coordinates of the intersection with the mesh triangle, $V_0$, $V_1$ and $V_2$ are the vertices of a triangle, *t* is the direction, *D* is the normalized ray trajectory, and *O* is the ray from the point of origin.

Finally, for each hemisphere, the indexes of the start ($Tri_{init}$) and end triangles ($Tri_{end}$) where the fiber intersects the mesh are obtained, as well as the coordinates of the two exact points of the initial ($Point_{init}$) and final ($Point_{end}$) intersection.

**Stage 4: cluster labeling**

This stage performs an automatic labeling of all the clusters based on the cortical regions they connect, by using a cortical ROI atlas. For testing, we use the Desikan–Killiany atlas [39], consisting of 35 regions (gyri) per hemisphere. We use the cortical meshes, containing a list of vertices and triangles, and a labeling file, containing the cortical region label of each mesh vertex.

**Fig. 14:**
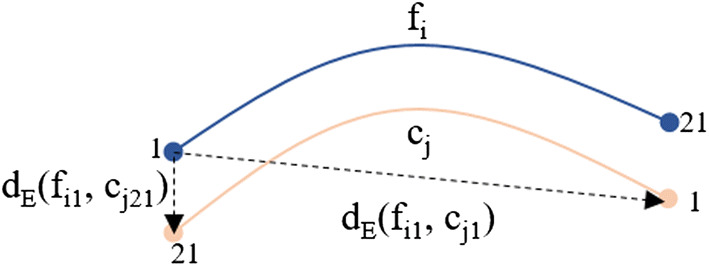
Fiber bundle alignment with respect to its corresponding bundle centroid. The Euclidean distance ($d_E$) is calculated between the first point of the fiber ($f_{i1}$) and both end points of the centroid ($c_{i1}$ to $c_{j21}$). If $d_E(f_{i1}, c_{j1})$ > $d_{E}(f_{i1}, c_{j21})$, the fiber is reversed

First, for each cluster, each fiber is labeled according to the triangle of the mesh that the fiber intersects, based on the region labels of the triangle vertices. The labeling of each triangle is defined as the most repeated label between its three vertices (see Fig. 13-(4.1.)). Next, the fibers require to be aligned, since, in a tractography dataset, there is no unique direction and fibers can be stored in direct or inverse direction. Since after the clustering the fibers are grouped on compact clusters, these can be aligned so that the starting and ending points have the same orientation in a cluster (see Fig. 13-(4.2.)). Hence, the fibers of a cluster are oriented based on the cluster centroid. To perform the alignment, we verify if the fibers are inverted with respect to the centroid. We denote $f_i$ as the fiber *i* of the bundle, with $i = 1, ..., n$, and the centroid of the bundle as $c_j$, with $j = 1, ..., m$. Then, the Euclidean distance ($d_E$) is calculated between the first point of the fiber ($f_{i1}$) and both endpoints of the centroid ($c_{i1}$ to $c_{j21}$). If $d_E(f_{i1}, c_{j1})$ > $d_{E}(f_{i1}, c_{j21})$, the fiber is inverted by flipping its fiber points (Fig. 14).

Next, each cluster (or bundle) is labeled according to the most connected regions. For each bundle, the labels of both bundle extremities, i.e., the beginning and end of each bundle, are processed separately. The most common label (*mcl*) for bundle start (${mcl_{init}}$) and end (${mcl_{end}}$) is determined and used to name each bundle, with format ${mcl_{init}}$-${mcl_{end}}$ (see Fig. 13-(4.3.)). For instance, a bundle connecting the postcentral and precentral anatomical regions will have the label *PoC-PrC*. Note that several bundles may connect the same pair of anatomical regions (gyri), as each cluster extremity only intersects a portion of a gyrus. Then, an order is assigned to each pair of bundles defined by the index of the regions in the cortical region label file. For example, *PrC* has index 24 and *PoC* has index 22, then, the bundle is named as *PoC-PrC*. Subsequently, bundles with inverted names are flipped. For example, all the bundles labeled with *PrC-PoC* are inverted and named as *PoC-PrC*. Finally, as several bundles may have the same name, but connecting different specific sub-regions of the gyri. These are labeled with an extra index, indicating the relative position according to an axis in the brain in MNI space. The intersection points of all the bundle centroids in a gyrus are sorted based on a spatial coordinate (*x*, *y* or *z*), in ascending or descending order. By default the order is ascending according to *y* axis, i.e., from the bottom up.

### Inter-subject labeling

In this section, two methods are presented to obtain automatic group-wise bundle labels of superficial white matter bundles, among the subjects of a population. Intra-subject labeling, presented in the previous section, labeled the bundles of a subject based on the connected brain regions individually, and an order based on the coordinates, producing a certain similarity between the subjects’ bundles. However, this was not the main objective of the intra-subject labeling method and the correspondence between subjects can be improved by applying inter-subject methods. The methods used to perform this processing are a matching algorithm and a clustering algorithm. To apply these methods, the tractography datasets need to be in a common space. All subjects were aligned to Talairach space using the affine transformation of the database, and then a rigid transformation to MNI space. Both methods use a maximum distance threshold to define the similarity of bundles across subjects. The inter-subject labeling renames all the bundles according to the correspondence found in the analyzed group of subjects. The part of the name related to the connected cortical regions is kept, but the index is assigned again to all the bundles. Bundles found similar in several subjects will have the same label.

**Matching algorithm for inter-subject labeling**

The aim of this step is to apply a matching [40] for finding a correspondence between similar bundles in the different subjects. Bipartite matching algorithms find correspondence between pairs of elements from two distinct sets. These algorithms are based on graph theory to find connections in two sets of vertices, where vertices in one set must match with vertices in the other set [41].

A well-known algorithm for a bipartite matching problem is the *Hungarian* algorithm, that solves the minimum weight matching, i.e., the minimum distance between vertices from the two sets, *A* and *B* [25]. Being *V* the number of vertices from the two sets, the algorithm receives a matrix *M*, containing the distances between the vertices from the two sets. In our application, *V* is the total number of bundles from a pair of subjects and matrix *M* contains the distances between the bundle centroids from the two subjects, being one set represented in the rows, and the other in the columns. The original algorithm performs a perfect matching, i.e., each vertex (or bundle) in set *A* is matched with a vertex in set *B*, which requires an equal number of vertices in both sets and produces a square matrix *M*. Our problem presents a different number of vertices in each set, as different number of bundles are found in each subject. Hence, we used an adapted algorithm that performs the analysis over non-squared matrices and leaves unmatched the most dissimilar elements.

More formally, each element *M*[*i*, *j*] in matrix *M* represents the distance between bundle *i* of set *A* (subject *A*) and bundle *j* of set *B* (subject *B*), being the cost of matching between the two vertices. The result is an assignment of the elements of set *A* with set *B* by using the minimum assignment cost. The distance used is the minimum average direct-flip distance (MDF) between two pairs of fibers [26] (Eq. 2), a distance commonly used for tractography fiber comparison. This distance calculates the mean Euclidean distance between corresponding points of a pair of centroids or fibers. To be used, fibers are resampled with a defined number of *K* equidistant points (21 in our case). Since fibers can be ordered in memory in opposite directions, the distance must be calculated with fibers in both directions (direct and flipped order), and then the minimum value must be selected, which will correspond to the correct order.

2 $$\begin{aligned} d_{\text {direct}}(a,b)&= d(a,b) = \frac{1}{K} \sum \limits _{i=1}^{K} |a_i - b_i| \nonumber \\ d_{\text {flipped}}(a,b)&= d(a,b^F) = d(a^F,b) \nonumber \\ MDF(a,b)&= \text {min}(d_{\text {direct}}(a,b), d_{\text {flipped}}(a,b)). \end{aligned}$$

The Hungarian algorithm has a complexity of ${\mathcal {O}}(V^3)$, however, as we perform the analysis separately for each pair of anatomical regions, the analyzed datasets are small with low execution time.

The matching algorithm applied to inter-subject bundle labeling first performs a bundle pre-processing. For each subject, previously labeled bundles with the intra-subject labeling, are separated into different groups depending on the pair of anatomical regions they connect. Then, for each region a map is created, whose key is the subject and the value is a list of the bundles that belong to the subject and region. For instance, for region *PoC-PrC* the bundles for *Subject001*, will be stored in the key-value pair: *Subject001: [bundle0, bundle1, ..., bundleN]*. Next, the algorithm consists of four steps:

- *Step 1*. Once the maps of all the regions are obtained, the bundles of each region are processed sequentially. First, the subjects are ordered from highest to lowest, based on the number of bundles they contain. For each bundle, its centroid is calculated using the mean of the streamline point coordinates.
- *Step 2*. The analysis begins with the first subject on the list as a reference subject. This subject is compared with each of the following subjects using the *Hungarian* algorithm, receiving as input the distance matrix. This returns a matching based on the distance of one bundle centroid with another. The *Hungarian* algorithm receives as input the matrix of distances, which are calculated using the MDF distance (Equation 2) between all the bundle centroid pairs of all subjects. For each bundle, the algorithm returns the bundle that best matches it, according to the solution of the minimization problem. However, the distance between a pair of bundles could be higher, hence, the method evaluates all the distances between the matched bundle centroids and only keeps the pairs of bundles in which distances do not exceed the established maximum distance threshold. This avoids the assignment of distant bundles, leaving them unassigned. Bundles that match each other are labeled with the same indexes, based on the label of the reference subject. For example, for two corresponding bundles, they would be called *PoC-PrC_0* even if they are from different subjects.
- *Step 3*. Two cases can happen with unassigned bundles: *(i) Bundles of the reference subject*. They are not similar to any other bundle in the dataset and they are labeled with a new index. *(ii) Bundles of the remaining subjects*. The bundles are stored. In the iteration in which the subject is taken as a reference, comparisons are made again with the rest of the subjects.
- *Step 4*. Repeat *Step 2* with the unassigned bundles of the following subjects, taking as reference the next subject in the list with unassigned bundles.

**Fig. 15:**
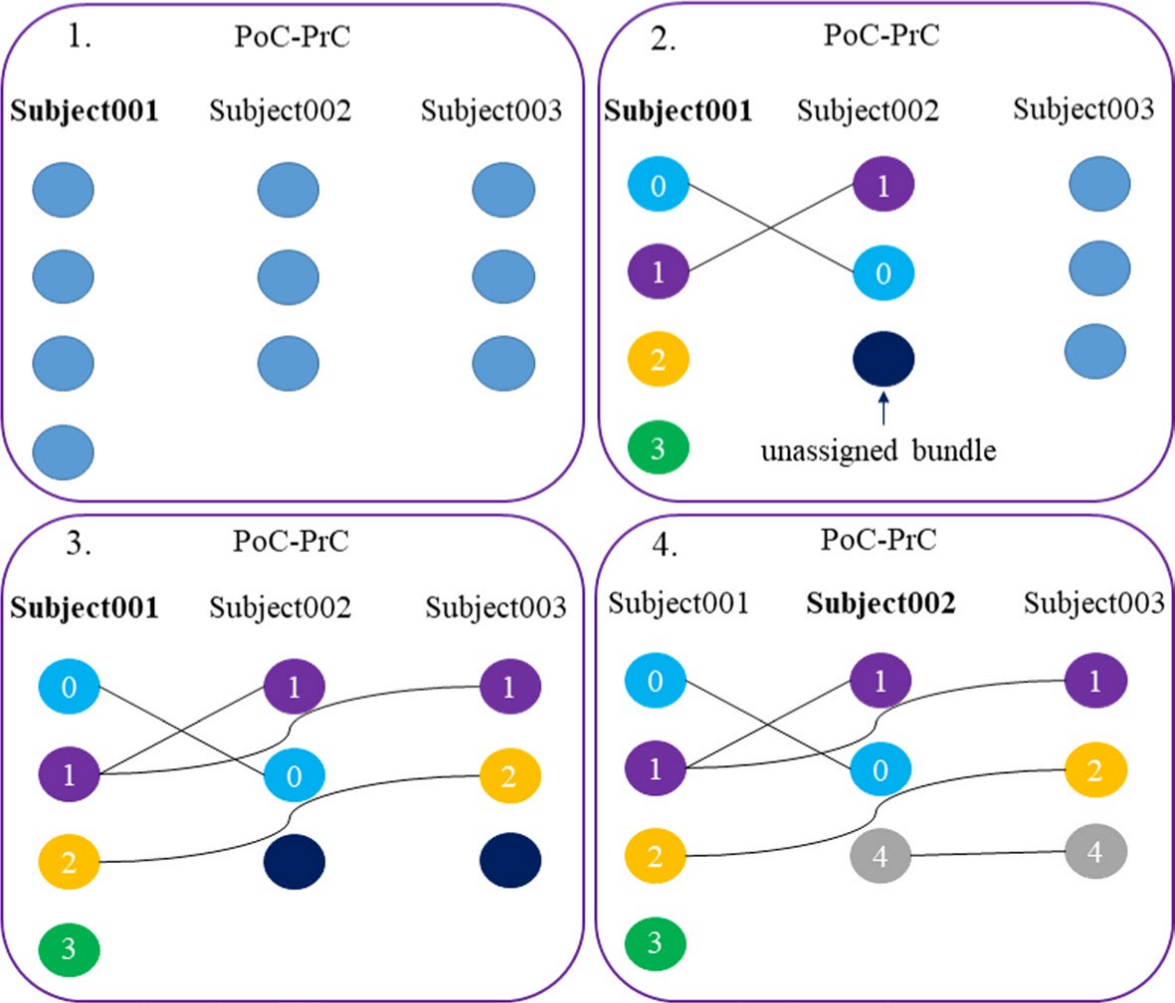
Schematics of the Hungarian algorithm for inter-subject labeling of bundles connecting PoC-PrC regions. First, the bundles are ordered from highest to lowest number of bundles. Second, the reference subject, *Subject001*, is compared to *Subject002*, leaving unassigned bundles. Third, it continues comparing to the rest of the subjects. Finally, the reference passes to the next subject with unassigned bundles, *Subject002* and these are compared with the rest of the subjects. This process is repeated until all subjects are analyzed

Figure 15 shows an example scheme of the algorithm for three subjects and the bundles connecting *PoC-PrC* regions. Each circle corresponds to a bundle. First, the subjects are ordered from highest to lowest number of bundles (see Fig. 15-(1.)). Second, *Subject001* that is being compared with the rest is the reference. It is compared with the *Subject002* and does match only the first two bundles, leaving an unassigned bundle in *Subject002*, which will be saved for later comparison (see Fig. 15-(2.)). Third, *Subject001* continues to be compared with the remaining subjects, in this case, with *Subject003*, which leads to the matching of bundles 1 and 2. In *Subject003* there remains an unassigned bundle (see Fig. 15-(3.)). Finally, once *Subject001* is compared with all subjects, the reference subject becomes the next one, in this case, *Subject002*. Then, unassigned bundles are compared, for example, *Subject002* is compared with *Subject003* and the two unassigned bundles are matched (see Fig. 15-(4.)). The bundle with the highest reproducibility in the example is the 1, since it is present in all subjects, and would be named as *PoC-PrC_1*, according to the label of the first reference subject of the bundle.

We used the implementation of the Hungarian algorithm available at scipy library [42].

**Clustering algorithm for inter-subject labeling**

Clustering is an unsupervised classification method, which groups similar elements into subsets called clusters. Each cluster is made up of elements that have similar characteristics, however, the elements of each cluster are different from each other [43].

The clustering method used to group the clusters is a well-known fiber clustering algorithm called *QuickBundles (QB)* [26, 44]. *QB* is a clustering method specialized in grouping white matter fibers from tractography datasets quickly and with good quality. This unsupervised clustering algorithm groups the fibers into clusters, without recalculating the clusters, like classical methods such as K-means. The algorithm uses a distance threshold to define whether a new fiber will be assigned to the closest cluster or will start a new cluster. The algorithm has a single parameter, which is the minimum average direct-flip distance (MDF) between two pairs of fibers. It is one of the fastest methods that exist today, with runtime ${\mathcal {O}}(N^2)$, being *N* the size of the dataset.

Before applying *QB*, we apply the same bundle pre-processing as for the matching, to create a map for each pair of regions, with the bundles of each subject. Next, the *QB* algorithm is performed sequentially to each pair of regions. For each pair of regions and all the subjects, the centroids of all clusters are calculated. The algorithm is applied to the complete set of clusters, i.e., from all subjects for the pair of regions. Once the inter-subject clusters are obtained, all intra-subject clusters belonging to the same inter-subject cluster are labeled with the same label. If several clusters of the same subject belong to the same inter-subject cluster, they are merged.

**Fig. 16:**
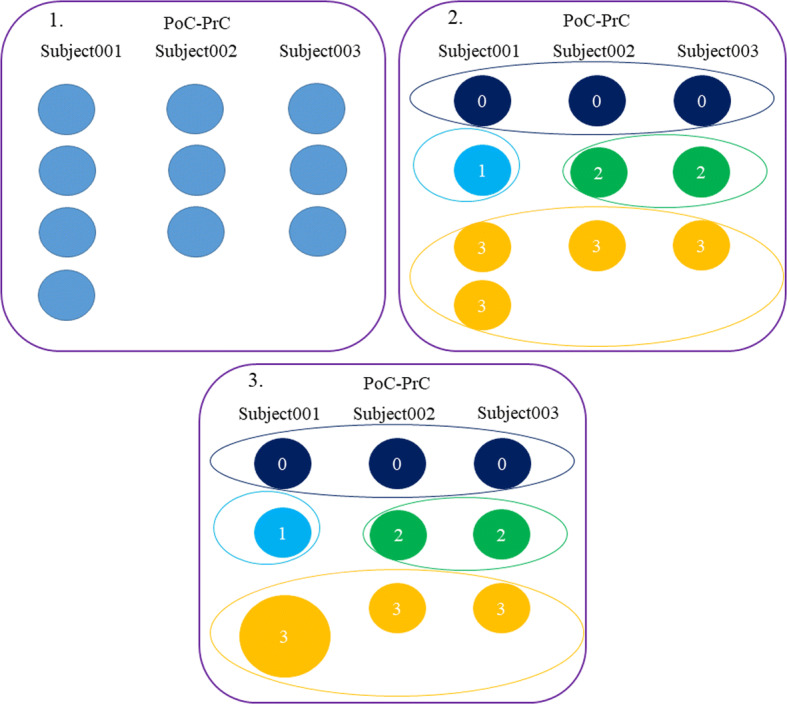
Schematics of the QB algorithm for labeling inter-subject bundles for PoC-PrC regions. First, the cluster centroids are computed. Second, *QB* is applied to all the intra-subject clusters, to obtain inter-subject clusters. Bundles belonging to an inter-subject cluster are labeled using the same name. Finally, clusters of the same subject that belong to the same inter-subject cluster are merged

Figure 16 shows an example scheme for *QB* application to three subjects on the *PoC-PrC* regions. First, it starts with the computation of all the cluster centroids. Unlike matching, in this case, it is not necessary that the clusters are ordered (see Fig. 16-(1.)). Second, the *QB* method is applied to all clusters, generating inter-subject clusters. Bundles within inter-subject clusters are labeled with the same name (see Fig. 16-(2.)). Finally, the clusters of a subject that are in the same inter-subject clusters are merged (see Fig. 16-(3.)). The clusters with the highest reproducibility are 0 and 3 since they appear in all subjects, whose tags would be: *PoC-PrC_0* and *PoC-PrC_3*. In addition, there may be some loose cluster, which will be individually labeled with another index.

## Statistical analysis

Histograms have been used to evaluate the reproducibility of inter-subject bundles (clusters) in terms of the number of subjects in where they are found, for the two tested methods and different distance thresholds. The greater the number of subjects, the more reproducible the bundle is. Another histogram displays the number of inter-subject bundles obtained for different inter-cluster distance ranges. The greater the number of clusters with a lower distance, the more precise the classification of the method is.

On the other hand, heatmaps were created to visually evaluate the reproducibility and variability of the bundles in the different subjects. First, the presence or absence of a bundle in a subject can be observed. Also, the normalized number of fibers is displayed using a heat colormap. The stronger the color indicates a higher number of fibers in the bundle.

Simple averages were used to compute the execution times over the different subjects.

## Data Availability

The datasets used and/or analyzed during the current study are available from the corresponding author upon request.

## Abbreviations

FA: Fractional anisotropy
SWM: Superficial white matter
DWM: Deep white matter
WM: White matter
QB: QuickBundles
PoC: Postcentral
PrC: Precentral
SM: Supra-marginal
RMF: Rostral middle frontal
SF: Superior frontal
SP: Superior parietal
IP: Inferior parietal
Tr: Pars triangularis
dMRI: Diffusion-weighted magnetic resonance imaging
MRI: Magnetic resonance imaging
HARDI: High angular resolution diffusion imaging
HCP: Human Connectome Project
ROI: Region of interest
MDF: Minimum average direct-flip

## Footnotes

1 brainvisa.info.

2 dsi-studio.labsolver.org.

## References

[1] Le Bihan D, Iima M (2015). Diffusion magnetic resonance imaging: what water tells us about biological tissues. PLoS Biol.

[2] Basser PJ, Mattiello J, LeBihan D (1994). Estimation of the effective self-diffusion tensor from the NMR spin echo. J Magn Reson Series B.

[3] Basser PJ, Pajevic S, Pierpaoli C, Duda J, Aldroubi A (2000). In vivo fiber tractography using DT-MRI data. Magn Reson Med.

[4] Catani M, De Schotten MT (2008). A diffusion tensor imaging tractography atlas for virtual in vivo dissections. Cortex.

[5] Martino J, De Witt Hamer PC, Vergani F, Brogna C, de Lucas EM, Vázquez-Barquero A, García-Porrero JA, Duffau H (2011). Cortex-sparing fiber dissection: an improved method for the study of white matter anatomy in the human brain. J Anat.

[6] Maier-Hein KH, Neher PF, Houde J-C (2017). The challenge of mapping the human connectome based on diffusion tractography. Nat Commun..

[7] Catani M, Dell’Acqua F, Vergani F, Malik F, Hodge H, Roy P, Valabregue R, De Schotten MT (2012). Short frontal lobe connections of the human brain. Cortex.

[8] Wassermann D, Makris N, Rathi Y, Shenton M, Kikinis R, Kubicki M, Westin C-F (2016). The white matter query language: a novel approach for describing human white matter anatomy. Brain Struct Funct.

[9] O’Donnell LJ, Westin C-F (2007). Automatic tractography segmentation using a high-dimensional white matter atlas. IEEE Trans Med Imaging.

[10] O’Donnell LJ, Suter Y, Rigolo L, Kahali P, Zhang F, Norton I, Albi A, Olubiyi O, Meola A, Essayed WI (2017). Automated white matter fiber tract identification in patients with brain tumors. NeuroImage Clin.

[11] Garyfallidis E, Côté M-A, Rheault F, Sidhu J, Hau J, Petit L, Fortin D, Cunanne S, Descoteaux M (2018). Recognition of white matter bundles using local and global streamline-based registration and clustering. NeuroImage.

[12] Guevara P, Duclap D, Poupon C, Marrakchi-Kacem L, Fillard P, Le Bihan D, Leboyer M, Houenou J, Mangin J-F (2012). Automatic fiber bundle segmentation in massive tractography datasets using a multi-subject bundle atlas. NeuroImage.

[13] Labra N, Guevara P, Duclap D, Houenou J, Poupon C, Mangin J-F, Figueroa M (2017). Fast automatic segmentation of white matter streamlines based on a multi-subject bundle atlas. Neuroinformatics.

[14] Vázquez A, López-López N, Labra N, Figueroa M, Poupon C, Mangin J-F, Hernández C, Guevara P. Parallel Optimization of Fiber Bundle Segmentation for Massive Tractography Datasets. In: 2019 IEEE 16th International Symposium on Biomedical Imaging (ISBI 2019); 2019. pp. 178–181. IEEE.

[15] Ros C, Güllmar D, Stenzel M, Mentzel H-J, Reichenbach JR (2013). Atlas-guided cluster analysis of large tractography datasets. PLoS ONE.

[16] Guevara M, Guevara P, Román C, Mangin J-F (2020). Superficial white matter: a review on the dMRI analysis methods and applications. NeuroImage.

[17] Guevara M, Román C, Houenou J, Duclap D, Poupon C, Mangin J-F, Guevara P (2017). Reproducibility of superficial white matter tracts using diffusion-weighted imaging tractography. NeuroImage.

[18] Román C, Guevara M, Valenzuela R, Figueroa M, Houenou J, Duclap D, Poupon C, Mangin J-F, Guevara P (2017). Clustering of whole-brain white matter short association bundles using HARDI data. Front Neuroinform.

[19] Meynert T. Psychiatry: a clinical treatise on diseases of the fore-brain based upon a study of its structure, functions, and nutrition. The Anatomy, Physiology, and Chemistry of the Brain. 1885.

[20] Tatsuya J, Seiichiro H, Tatsuya Y, Keiko K, Yasuo I, Atsushi Y (2020). White matter dissection and structural connectivity of the human vertical occipital fasciculus to link vision-associated brain cortex. Sci Rep.

[21] O’Donnell LJ, Golby AJ, Westin C-F (2013). Fiber clustering versus the parcellation-based connectome. NeuroImage.

[22] Zhang F, Wu Y, Norton I, Rigolo L, Rathi Y, Makris N, O’Donnell LJ (2018). An anatomically curated fiber clustering white matter atlas for consistent white matter tract parcellation across the lifespan. NeuroImage.

[23] Labeling. https://github.com/andvazva/Labeling.git/. Accessed 25 Apr 2020

[24] Schmitt B, Lebois A, Duclap D, Guevara P, Poupon F, Rivière D, Cointepas Y, LeBihan D, Mangin J-F, Poupon C. CONNECT/ARCHI: an open database to infer atlases of the human brain connectivity. In: ESMRMB; 2012.

[25] Frank A (2005). On Kuhn’s Hungarian method-a tribute from Hungary. Naval Res Logist.

[26] Garyfallidis E, Brett M, Correia MM, Williams GB, Nimmo-Smith I (2012). Quickbundles, a method for tractography simplification. Front Neurosci.

[27] Guevara P, Duclap D, Marrakchi-Kacem L, Rivière D, Cointepas Y, Poupon C, Mangin J. Accurate tractography propagation mask using T1-weighted data rather than FA. In: Proceedings of the International Society of Magnetic Resonance in Medicine; 2011. p. 2018

[28] Zhang Y, Zhang J, Oishi K, Faria AV, Jiang H, Li X, Akhter K, Rosa-Neto P, Pike GB, Evans A, Toga AW, Woods R, Mazziotta JC, Miller MI, van Zijl PCM, Mori S (2010). Atlas-guided tract reconstruction for automated and comprehensive examination of the white matter anatomy. NeuroImage.

[29] Mangin J-F, Lebenberg J, Lefranc S, Labra N, Auzias G, Labit M, Guevara M, Mohlberg H, Roca P, Guevara P (2016). Spatial normalization of brain images and beyond.

[30] BrainVISA. http://brainvisa.info/web/index.html Accessed 25 Apr 2020

[31] Descoteaux M, Angelino E, Fitzgibbons S, Deriche R (2007). Regularized, fast, and robust analytical Q-ball imaging. Magn Reson Med.

[32] Perrin M, Poupon C, Cointepas Y, Rieul B, Golestani N, Pallier C, Rivière D, Constantinesco A, Le Bihan D, Mangin J-F. Fiber tracking in q-ball fields using regularized particle trajectories. In: Biennial International Conference on Information Processing in Medical Imaging, Springer; 2005. p. 52–63. 10.1007/11505730_5 17354684

[33] FreeSurfer. https://surfer.nmr.mgh.harvard.edu/fswiki. Accessed 25 Apr 2020

[34] Vázquez A. Método eficiente de clustering de fibras cerebrales basado en distribución de puntos. Master’s thesis in Computer Science, Universidad de Concepción, Concepción; 2019.

[35] Sanchez A, Hernández C, Poupon C, Mangin J-F, Guevara P. Clustering of tractography datasets based on streamline point distribution. In: International Society of Magnetic Resonance in Medicine Conference; 2018. ISMRM 2018

[36] Guevara P, Poupon C, Rivière D, Cointepas Y, Descoteaux M, Thirion B, Mangin J-F (2011). Robust clustering of massive tractography datasets. NeuroImage.

[37] Silva F, Guevara M, Poupon C, Mangin J-F, Hernández C, Guevara P. Cortical surface parcellation based on graph representation of short fiber bundle connections. In: 2019 IEEE 16th International Symposium on Biomedical Imaging (ISBI 2019); 2019. p. 1479–1482. IEEE

[38] Möller T, Trumbore B. Fast, minimum storage ray/triangle intersection. In: ACM SIGGRAPH 2005 Courses; 2005. p. 7. ACM.

[39] Desikan RS, Ségonne F, Fischl B, Quinn BT, Dickerson BC, Blacker D, Buckner RL, Dale AM, Maguire RP, Hyman BT (2006). An automated labeling system for subdividing the human cerebral cortex on MRI scans into gyral based regions of interest. NeuroImage.

[40] Monge AE, Elkan C (1996). The field matching problem: algorithms and applications. KDD.

[41] Wilson RJ (1986). Introduction to graph theory.

[42] Solve the Linear Sum Assignment Problem. https://docs.scipy.org/doc/scipy-0.18.1/reference/generated/scipy.optimize.linear_sum_assignment.html. Accessed 25 Apr 2020

[43] Xu R, Wunsch DC. Survey of clustering algorithms; 2005. 10.1109/TNN.2005.845141 15940994

[44] Tractography Clustering with QuickBundles. https://dipy.org/documentation/1.0.0./examples_built/segment_quickbundles/. Accessed 25 Apr 2020

